# Supplementary material for: Circulating tumor DNA reveals mechanisms of lorlatinib resistance in patients with relapsed/refractory ALK-driven neuroblastoma
Source: Nat Commun. 2023 May 5;14:2601. doi: 10.1038/s41467-023-38195-0 (PMC10163008; doi:10.1038/s41467-023-38195-0)
Supplement: Supplementary file 1 — Supplementary Information [file 41467_2023_38195_MOESM1_ESM.docx]

**Supplementary Data**

**Circulating tumor DNA reveals mechanisms of lorlatinib resistance in patients with relapsed/refractory ALK-driven neuroblastoma**

Esther R Berko^1,3,15^ , Gabriela M Witek^1,2,4,15^, Smita Matkar^1,15^, Zaritza O. Petrova^5,6,15^, Megan A. Wu^5,6^, Courtney M. Smith^5,6^, Alex Daniels^1^, Joshua Kalna^1^, Annie Kennedy^1^, Ivan Gostuski^4^, Colleen Casey^1^, Kateryna Krytska^1^, Mark Gerelus^1^, Dean Pavlick^7^, Susan Ghazarian^8,9^, Julie R. Park^10^, Araz Marachelian^8,9^, John M. Maris^1,2^, Kelly C. Goldsmith^11,12,13^, Ravi Radhakrishnan^4,14^, Mark A. Lemmon^5,6^ and Yaël P. Mossé^1,2^

**Affiliations**

^1^ Division of Oncology and Center for Childhood Cancer Research; Children’s Hospital of Philadelphia; Philadelphia, Pennsylvania, USA

^2^ Perelman School of Medicine at the University of Pennsylvania; Philadelphia, PA, USA

^3^ Division of Pediatric Hematology and Oncology, Schneider Children’s Medical Center, Petach Tikva, Israel; Faculty of Medicine, Tel Aviv University, Tel Aviv, Israel

^4^ Department of Bioengineering, University of Pennsylvania, Philadelphia, PA, USA

^5^ Department of Pharmacology, Yale University School of Medicine, New Haven, CT, USA

^6^ Yale Cancer Biology Institute, Yale University, West Haven, CT, USA

^7^ Foundation Medicine, Inc. Cambridge, MA, USA

^8^ Cancer and Blood Disease Institute, Children’s Hospital Los Angeles, Los Angeles, California, USA

^9^ Keck School of Medicine, University of Southern California, Los Angeles, California, USA

^10^ St. Jude Children’s Research Hospital, Memphis, TN, USA

^11^ Aflac Cancer and Blood Disorders Center, Children’s Healthcare of Atlanta, Atlanta, Georgia, USA

^12^ Winship Cancer Institute, Emory University School of Medicine, Atlanta, Georgia, USA

^13^ Seattle Children’s Hospital, Seattle, Washington, USA

^14^ Department of Chemical and Biomolecular Engineering, University of Pennsylvania, Philadelphia, PA, USA

^15^ These authors contributed equally

Yaël P. Mossé, MD

Children's Hospital of Philadelphia,

Perelman School of Medicine at the University of Pennsylvania,

Division of Oncology,

3501 Civic Center Blvd

CTRB 9006

Philadelphia, PA 19104

Tel: 215 590-0965

Email: [mosse@chop.edu](mailto:mosse@chop.edu)

Mark A. Lemmon, PhD

Yale Cancer Biology Institute,

Yale University West Campus,

ABC 301, 840 West Campus Drive,

PO Box 27400

West Haven, CT 06516

Tel:  203-785-6081

Email: [mark.lemmon@yale.edu](mailto:mark.lemmon@yale.edu)

**Supplementary Data Fig. 1. Enrollment *ALK* VAF and Curie Score**

The detectable *ALK* VAF at enrollment did not directly correlate with disease burden as measured by total Curie score. VAF, Variant allele frequency. Source data are provided as a Source Data file.

**Supplementary Data Fig. 2. Total number of ctDNA samples**

Barplot showing the distribution of the total number of ctDNA samples obtained for the 46 patients in the study cohort. ctDNA, circulating tumor DNA

**Supplementary Data Fig. 3. Correlation between VAF and CTF**

ALK VAF (blue dashed line) and CTF (red solid line) values on the y-axis with course of treatment on the x-axis show that the estimated proportion of detectable ctDNA in the cell free DNA CTF followed the same trend as the *ALK* VAF. ctDNA, circulating tumor DNA. CTF, comprehensive tumor fraction. VAF, variant allele frequency. Source data are provided as a Source Data file.

**Supplementary Data Fig. 4. Enrichment of off-target mutations with lorlatinib resistance**

For 4 patients in which circulating *ALK* VAF did not correlate with disease response, we observed enrichment of alternative off-target mutations that could be responsible for mediating lorlatinib resistance. Patients 7, 27 and 37 had no response to lorlatinib and progressed at timepoint 2. Patient 7 harbored *MET* and *BRAF* amplifications as well as the *TP53* mutations shown in **a**. **b**, Patient 15 had a transient response at course 2 and then progressed, with increase in the VAF of the detectable *TP53* variant. Patients 27 (**c**) and 37 (**d**) harbored *NF1* and *HRAS* mutations respectively. VAF, variant allele frequency. Source data are provided as a Source Data file.

**
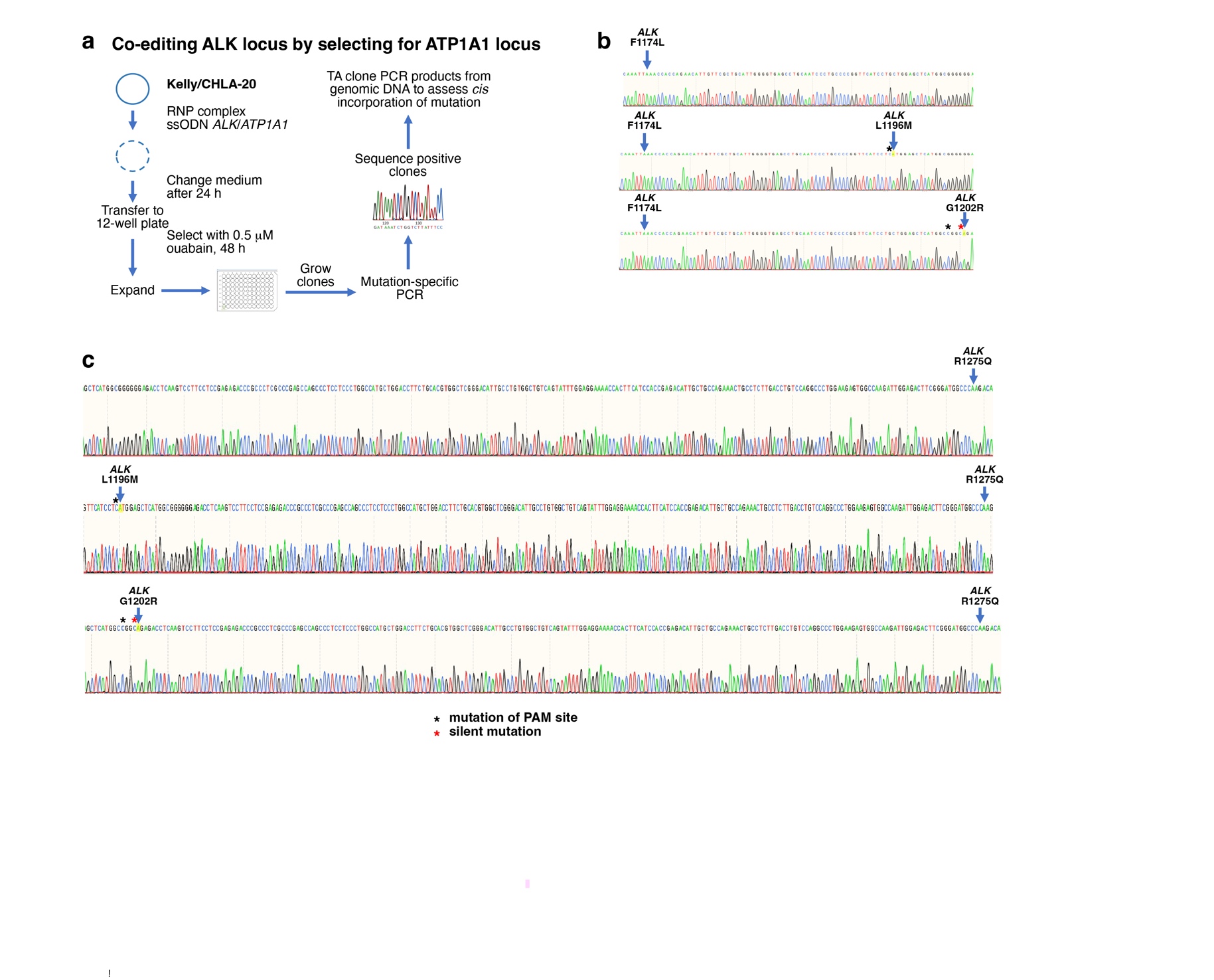
**

**Supplementary Data Fig. 5. CRISPR-CAS-driven targeted co-editing of *ALK* and *ATP1A1* locus**

**a**, Schematic showing experimental strategy for introducing mutations at *ALK* and *ATP1A1* locus. **b**, Genomic DNA Sanger sequencing results showing L1196M and G1202R mutations were introduced into the F1174L-mutated *ALK* gene of the Kelly cell line in *cis*. **c.** Sanger sequencing results for cDNA from the CHLA-20 cell line showing that the G1202R mutation and L1196M mutation were introduced in *cis* into the R1275Q-mutated *ALK* gene.

**__**

**Supplementary Data Fig. 6. Biochemical analysis of crizotinib inhibition and the ΔD1276-R1279InsE variant**

**a**, **b** Crizotinib competition curves for purified F1174L (**a**) and R1275Q (**b**) ALK-TKD variants. In **a**, IC_50_ values were fit for F1174L-mutated ALK-TKD (open red circles, solid red curve: IC_50_ = 40 ± 20 nM), F1174L/L1196M (medium red open diamonds, dashed curve: IC_50_ = 150 ± 51 nM) and F1174L/G1202R (dark red open squares, dotted curve: IC_50_ = 437 ± 175 nM). In **b**, IC_50_ values were fit for R1275Q ALK-TKD (open blue circles, solid blue curve: IC_50_ = 38 ± 24), R1275Q/L1196M (medium blue diamonds, dashed curve: IC_50_ = 155 ± 89 nM) and R1275Q/G1202R (dark blue squares, dotted curve: IC_50_ = 209 ± 50 nM). Data are plotted as the mean ± SD of three biological replicates, each performed in technical triplicate. IC_50_ values are listed in Supplementary Table 1**.** **c**, Initial velocities for purified ALK-TKD harboring the ΔD1276-R1279InsE mutation using the ^32^P incorporation method previously described^22^ to assess phosphotransfer with 50 nM unphosphorylated ALK-TKD, 2 mM ATP and the noted concentrations of a peptide mimic of the ALK activation loop with sequence: biotin-ARDIYRASYYRKGGCAMLPVK (CanPeptide). This is representative of three biological repeats, and is quantitated in **d** to estimate *k*_cat_ for this variant as 71 ± 17 min^-1^, compared with 9 min^-1^ for wild type, 365 min^-1^ for F1174L, and 119 min^-1^ for R1275Q^22^. Source data are provided as a Source Data file.

**Supplementary Data Fig. 7. Structural overview of the ALK binding pocket and lorlatinib docking poses**

The color designations for ALK variants in this figure are as follows: F1174L (red) F1174L/G1202R (dark red), F1174L/L1196M (medium red), R1275Q (blue), R1275/G1202R (dark blue), R1275Q/L1196M (medium blue), G1202R (gold) F1245C (green). **a**-**c**, Overlay of best-fit docked lorlatinib (green) poses in the ATP-binding sites of wild type ALK-TKD (**a**, docking score = -11.208 kcal/mol), F1174L (**b**, docking score = -9.632 kcal/mol), and R1275Q (**c**, docking score = -11.477 kcal/mol). In each panel, the position of the bound lorlatinib is overlaid with that seen in the crystal structure of lorlatinib-bound wild type ALK-TKD (PDB ID: 4CLI^15^, and bound waters are shown as red spheres. **d**, Correlation of average lorlatinib docking scores from computational studies (vertical axis, in kcal/mol) and experimentally derived values of *K*_i_ (plotted as -log(*K*i)), for wild type ALK-TKD and the F1174L, F1245C, and R1275Q variants. Data are fit to a straight line with regression coefficient R2 of 0.859. **e**, Calculated values of solvent accessible surface area (SASA) in the binding pocket of ALK-TKD, showing a bimodal distribution in the size of the pocket for the G1202R variant (gold in both panels) and the F1174L/G1202R variants (dark red, in left panel), but not for the F1174L variant (red in left panel) of the R1275Q variants (blue and dark blue in right panel). The bimodal distribution of binding site SASA values indicates structural heterogeneity for the G1202R and F1174L/G1202R variants. **f**, Table of lorlatinib docking scores, as plotted in Figure 6b, suggesting that the resistance of the F1174L/G1202R variant is not likely to arise simply from impaired lorlatinib docking. The docking scores were generated with *n* of 10 independently run induced fit docking experiments. **g**, Best-fit docked lorlatinib (medium blue) pose for the R1275Q/L1196M variant (docking score = -9.885 kcal/mol), showing the preserved hydrogen bonding (blue dashed lines) interactions of the aminopyridine group with the backbone carbonyl of E1197 and the backbone amide of M1199. **h**, Lorlatinib (dark blue) docked in the binding site of R1275Q/G1202R (docking score = -10.374 kcal/mol). Source data are provided as a Source Data file.

**Supplementary Data Fig. 8. Modeling of lorlatinib inhibition of different ALK-TKD conformations**

The color designations for ALK variants in this figure are as follows: F1174L (red) F1174L/G1202R (dark red), F1174L/L1196M (medium red), R1275Q (blue), R1275/G1202R (dark blue), R1275Q/L1196M (medium blue). **a**, Modeled curves for competitive inhibition of ALK-TKD variants generated *in silico* for the F1174L variant and F1174L-containing compound mutations, as described in Methods and Supplementary Note 2. The modeled reaction contained 1 mM ATP and 5 nM ALK-TKD to mimic the experimental studies. Data are plotted as the calculated remaining concentration of ATP-bound ALK-TKD as lorlatinib concentration is increased, with IC_50_ corresponding to the lorlatinib concentration at which only 50% of the ALK-TKD is ATP-bound (and the remainder lorlatinib-bound). All curves were generated using the gate-open kinetic reaction model. The curves have the following designations for each variant: The F1174L (red circles, solid curve), F1174L/L1196M (medium red diamonds, dashed curve) and F1174L/G1202R (dark red squares, dotted curve). **b**, Calculation of lorlatinib inhibition of the F1174L/G1202R variant generated *in silico* with the kinetic model that allows gate closure (see Supplementary Data Fig. 9c), with the gate closed for different percentages of the reaction time: 0% (black), 10% (light blue), 33% (dark blue), 66% (red), 90% (pink). **c**,**d**, Fold change in IC_50_ (compared with the corresponding single mutation: F1174L or R1275Q) for each compound mutation. Closed bars represent computational results (comp), and open bars represent experimental results (exp), from the biochemical data presented in Figure 5 and Supplementary Table 1. **e**, Table of computed IC_50_ values for ALK-TKD variants in which the gate-closed model was either ignored (right-hand column) or included (middle column), with frequency of gate closure reflecting that seen for the given ALK-TKD variant in MD studies (see Figure 6f). Source data are provided as a Source Data file.

**Supplementary Data Fig. 9. Validation of lorlatinib docking and gate-closed kinetic models**

**a**,**b**, Docking of lorlatinib into wild type ALK-TKD with (**a**) and without (**b**) modeled water, overlaid with the crystal structure, PDB ID: 4CLI^15^. The crystal structure is represented by lorlatinib in green and remaining structures in grey. All modeled structures are colored blue. The lorlatinib pose for wild type ALK-TKD is slightly altered with modeled waters (**a**) compared to that without (**b**), and the docking score is reduced to -10.263 kcal/mol from -8.374 kcal/mol. **c**, Schematic representations of the two kinetic schemes used to model competitive inhibition of ALK-TKD with lorlatinib. The upper model allows the gate-closed conformation to be accessed (in G1202R/F1174L variant), whereas the lower does not: the gate remaining open. E1 refers to ALK-TKD in the gate-open conformation (when lorlatinib can bind to form E1L) and E2 refers to ALK-TKD in the gate-closed conformation, to which lorlatinib cannot bind. ATP can bind ALK-TKD in either conformation to generate E1A and E2A. See Supplementary Note 2 for details **d**, Lorlatinib docking pose clusters. Cluster 1 (green) represents the crystal structure configuration seen in PDB ID: 4CLI, and other clusters represent poses rotated as marked with respect to that pose. To calculate the final average docking score, the ALK-TKD conformation from MD simulations that yielded the lowest (best) docking score in Cluster 1 was identified for each variant, and docking runs performed on this conformation were analyzed. The resulting docking scores were averaged to obtain an arithmetic mean that is reported in Figure 6b. **e**, Graph of distance between the α carbon of R1202 and that of L1122 over the course of 100 ns of MD simulation of the F1174L/G1202R (dark red) variant (from 100-200 ns), showing how lorlatinib (green) clashes with R1202 and L1122 when the R1202Cα/L1122Cα distance falls below 8.9 Å. A histogram of lorlatinib docking scores (kcal/mol) corresponding to the ALK conformations derived at different points of this 100 ns MD run are shown at the bottom, with poor docking scores (>-6 kcal/mol) when the R1202Cα/L1122Cα distance falls below 8.9 Å (the gate-closed conformation) but remains favorable when the R1202Cα/L1122Cα distance is above this value (the gate-open conformation). Source data are provided as a Source Data file.

**Supplementary Note 1. Additional notes on clinical data.**

**Patient 2** was enrolled on dose level 1 (80% of adult RP2D) and harbored an *ALK* F1245V mutation (VAF = 43%) at enrollment. This patient acquired several additional *ALK* mutations upon disease progression at course 4 that may suggest novel F1245V-containing compound mutations associated with lorlatinib resistance. This patient also had multiple deleterious *TP53* mutations (R175H at 12.8%, E258D at 0.27%, A189G at 17.5%, A276P at 0.7%, G244C at 3%, T125M at 0.4%, as well as a *TP53* rearrangement with *TNFSF12*), plus a *TERT* promoter mutation (-124C>T at 18%), an *NF1* indel P2472fs* (VAF = 0.5%), and a *BRCA2* indel F2410fs* (VAF = 31%). This patient had a minor response to therapy, with a decrease in RECIST criteria and complete response of marrow disease after 2 courses of therapy. At this timepoint, ctDNA assessment detected all of the patient’s previously observed alterations (except the *BRCA2* indel and the *TP53* E258D mutation), but reported a decrease in VAF for the *ALK* F1245V, *TP53* A189G, *TP53* A276P, *TP53* R175H, *TERT* promoter, and *NF1* frameshift mutations. The patient subsequently progressed at course 4, and ctDNA analysis showed an increase in the *ALK* F1245V variant (VAF = 38%) with acquisition of several new *ALK* mutations: I1171T (VAF = 0.28%), R1192P (VAF = 0.13%) and L1196M (VAF = 0.79%). Analysis of individual sequencing reads showed that the *ALK* R1192P and L1196M mutations are in *trans* (*i.e.*, on different alleles). Since the I1171T and F1245V mutations are on separate reads due to chromosomal distance, it was not possible to determine if they co-occur on the same allele. As these *ALK* mutations were acquired, VAFs were reduced for some *TP53* mutations (T125M lost, R175H reduced to 7.86%, A189G reduced to 8%, and A276P reduced to 0.35%), but increased for others (G244C up to 4.66%). The *NF1* mutation was retained (VAF = 0.35%), and a new *PTPN11* G503A mutation (VAF = 0.6%) was detected.

**Patient 5** was enrolled on dose level 2 (equivalent to the adult RP2D) and harbored an *ALK* F1174L mutation (VAF = 43%) at enrollment, as well as *MYCN* amplification. This patient had a minor response after 2 courses of therapy, with stable disease on MIBG and decrease in RECIST criteria. The VAF for *ALK* F1174L remained essentially stable at 47%. The patient then had progressive disease at course 4, when ctDNA analysis showed an increase in the F1174L VAF to 92% with concurrent ALK amplification. The sample also revealed newly acquired *ALK* G1202R (VAF = 2.36%) and D1203N (VAF = 0.47%) mutations, each in *cis* with the F1174L variant but in *trans* with each other.

**Patient 13** was enrolled on dose level 3A and harbored an *ALK* R1275Q mutation (VAF = 63%) at enrollment as well as a *BRCA2* N863fs* mutation (VAF = 0.31%). This patient had stable disease, and ctDNA analysis at course 6 showed only a decrease in the *ALK* variant to 9.61%. At course 10, the patient still had stable disease, but ctDNA demonstrated an increase in VAF for the *ALK* R1275Q variant to 88%, along with acquisition of a new *ALK* G1202R mutation (VAF = 0.14%) plus reemergence of the *BRCA2* mutation (VAF = 0.77%). At course 12, the patient had clinically developed progressive disease on MIBG; ctDNA showed persistence of the *ALK* R1275Q mutation at 92%, an increase in VAF for the *ALK* G1202R mutation to 0.17%, persistence of the *BRCA2* mutation, and acquisition of a *BRAF* V600E mutation (VAF = 0.13%). Due to the genomic distance between the region that encodes R1275 and the position that encodes G1202 in *ALK*, we cannot determine if the two *ALK* mutations co-occur in *cis*, although it seems highly likely.

**Patient 14** was enrolled on dose level 3A, and harbored an *ALK* F1174C mutation (VAF = 23.5%) at enrollment along with a *CDKN2A* A102V mutation (VAF = 25.5%). This patient had a partial response to lorlatinib therapy at course 2 with complete resolution of MIBG and bone marrow disease and a decrease in RECIST criteria. Analysis of ctDNA at this timepoint showed a decrease in the *ALK* F1174C VAF to 0.16%. The clinical response persisted and ctDNA from course 4 showed no detectable variants. Unfortunately, after course 6, the patient had signs of clinical progression and was taken off therapy. A follow-up ctDNA sample showed an increase in VAF for the *ALK* F1174C mutation to 37%, as well as new acquisition of an *ALK* G1202R mutation in *cis* (VAF = 3.9%). This ctDNA sample also showed re-emergence of the *CDKN2A* variant (VAF = 8.32%) and a newly acquired *PTPN11* D61Y mutation (VAF = 0.91%).

**Patient 24** was enrolled on dose level 4A, and harbored an *ALK* F1174L mutation (VAF = 0.57%) at enrollment along with *MYCN* amplification. At course 2, this patient had stable disease with ctDNA showing persistence of the *ALK* F1174L variant at 0.37%. The patient developed progressive disease on MIBG at course 4, and ctDNA analysis showed an increase in VAF for the *ALK* F1174L variant to 8% as well as a newly acquired *ALK* L1196M mutation (VAF = 4.67%) in *trans*.

**Patient 25** was enrolled on dose level 4, and harbored an *ALK* F1174L mutation (VAF = 7%) at enrollment, along with MYCN amplification. This patient had clinically stable disease at course 2, and ctDNA showed an increase in the *ALK* variant to 15.47%. At course 4 the patient developed progressive disease on both MIBG and RECIST assessment, and ctDNA demonstrated an increase in VAF for the F1174L variant to 40.8% as well as a newly acquired *ALK* L1196M mutation in *cis* (VAF = 0.16%).

**Supplementary Note 2. Details of computational methods for assessing docking and lorlatinib competition.**

**I. Lorlatinib docking score analysis and comparison with experimental *K*_i_ values**

Subject to well appreciated caveats and limitations, molecular docking and the resulting docking score is viewed as an estimated correlator for binding energy of an inhibitor^1^. We employed induced fit molecular docking (IFD) to estimate changes in lorlatinib binding energies due to secondary *ALK* mutations. IFD was performed on 10 ALK-TKD conformations generated from our MD simulations as described in the Methods section of the manuscript. Each IFD run produced 0-18 docked lorlatinib poses per ALK-TKD conformation: some closely resembling the pose from the crystal structure (PDB ID: 4CLI), and some rotated in reference to that pose. The docked poses were classified into four clusters depending on the lorlatinib orientation (**Extended Data Fig. 7d**), where the set of poses matching the crystal structure orientation in PDB ID: 4CLI were assigned as Cluster 1. To calculate the final average docking score, the ALK-TKD conformation from MD simulations that yielded the lowest (best) docking score in Cluster 1 was identified for each variant, and docking runs performed on this conformation (with different random seeds) were analyzed. The resulting docking scores were averaged to obtain an arithmetic mean that is reported in **Figure 6b**. The Cheng-Prusoff equation^2^ was used to estimate experimental *K*_i_ values for lorlatinib for known ALK variants from previously reported IC_50_ values^3^, using **Equation S1**^4^

$K_{i}=\frac{{IC}_{50}}{(1+(\frac{[ATP]}{K_{M,ATP}}))}$ **Equation S1**

Given the logarithmic dependence of the binding free energy on *K*_I_ (see **Equations S2** and **S3)**, the computed average docking score was correlated with -log_10_(*K*_i_) (**Extended Data Fig. 5d)**. The resulting goodness of fit R^2^ value was found to be 0.86.

${K_{i}= e}^{\frac{{\Delta G}}{RT}}$, where ∆*G* is the binding free energy **Equation S2**

${{-log K_{i}}= {\Delta G}}$ **Equation S3**

**II. S****tructural analysis of gate-open and gate-closed ALK-TKD conformations**

Analysis of 100 ns MD simulations (n = 3) for each ALK variant was used to calculate the time that the ALK-TKD variants are present in the ‘gate-closed’ and ‘gate-open’ binding pocket conformations. To test our hypothesis that the gate is closed only in the G1202R systems, or more specifically that the lorlatinib binding pocket is occluded only in the G1202R variants, we subjected all variants in our study to the following analysis. The distance between the α carbons of R/G1202 and L1122 were calculated using the *gmx distance* tool from the GROMACS suite^5^. To assess the proportion of time ALK spends in the gate-open binding pocket conformation, the distance between different the L1122 and G/R1202R α carbons was calculated and correlated to lorlatinib binding, where binding is defined by the presence of lorlatinib in the binding pocket and a docking score below -6 kcal/mol. The distance between L1122:Cα and G/R1202:Cα showed a better correlation with lorlatinib binding than did distances between Cγ of L1122 and Cζ of G/R1202 or between Cα of L1122 and Cζ of G/R1202 (**Supplementary Table 2)**. Analysis of the trend in the distance between the L1122 and G/R1202 Cα atoms showed that separation distances below 8.9 Å prevent lorlatinib from binding to the pocket (**Extended Data Fig. 7e**). Specifically, the docking scores for lorlatinib binding to the gate-open conformation were found to be in the range of -10.53 to -12.03 kcal/mol, as plotted in **Extended Data Fig. 7e**; the gate-closed conformation did not bind lorlatinib (computed docking scores ranged from -2.92 to -4.91 kcal/mol). Applying the distance cut-off of 8.9 Å, we analyzed 300 ns of MD simulation for each ALK variant, and calculated the percent of the simulation time each variant spends in the gate-open drug-binding-competent conformation (**Figure 6f** in the main text).

Solvent accessible surface area (SASA) was calculated using the *gmx sasa* analysis tool in the GROMACS suite^5^, selecting for analysis residues located in the binding pocket based on the crystal structure of wild type ALK-TKD with bound lorlatinib (PDB ID: 4CLI^6^). Binding pocket residues were determined using the maestro selection tool for binding site residues; this tool identifies residues within 5 Å of the bound ligand – which in this case were: 1122-1130, 1148-1150, 1167, 1180, 1196-1203, 1206, 1249, 1253-1256, 1269-1273. These residues were used to calculate the aggregate SASA for each variant ALK structure as reported in **Extended Data Fig. 5e**, with characteristics summarized in **Supplementary Table 3** for the different ALK-TKD variants.

**III. Modeling of lorlatinib/ATP competition and inhibition**

A computational model of competitive binding of lorlatinib to ALK in the presence of excess ATP was used to validate our predicted resistance mechanisms, and to assess whether resistance arises primarily from a decrease in lorlatinib binding energy or occlusion of the drug-binding site in the gate-closed conformation. We modeled IC_50_ values as described below, and compared them with the experimental IC_50_ values reported in **Supplementary Table 1** of this paper. We defined our modeled lorlatinib IC_50_ value as the drug concentration at which 50% of the ALK-ATP complex is depleted in our model by out-competing ATP for the binding site – recapitulating the biochemical measurement (where IC_50_ represents the lorlatinib concentration that reduces activity by 50%).

In the first step, described below in more detail, rates of ATP association and dissociation for ALK-TKD were estimated from experimental *K*_M,ATP_ values measured in this study (**Section IV** below). Next, predicted association and dissociation rate constants for lorlatinib were estimated based on the calculated docking scores for each ALK variant, and calibrated using calculations and published experimental data for the F1174L and R1275Q single mutations (**Section V** below). We then calculated modeled IC_50_ values using the gate-open and gate-closed kinetic models summarized in **Extended Data Fig. 7c**, using a range of gate closing frequencies in the latter case. E1 represents ALK in the gate-open conformation, and E2 represents a conformation in which the binding pocket is occluded due by the arginine at position 1202. IC_50_ values were derived using the differential equation modeling tool, COPASI v. 4.27^7^. All COPASI files used to derive IC_50_ can be accessed via GitHub (<https://github.com/witekgabriela/StructuralStudy_ALKCompoundMutations>).

**IV. Estimation of ATP association and dissociation rate constants for inhibition model**

The ATP association rate constant (*k*_on,ATP_) for each ALK variant was estimated using the diffusion limited approximation given by **Equation S4**. This equation represents the fastest rate of collision between ALK-TKD and ATP, accounting for their sizes and the rate of diffusion of a ligand such as ATP in water. In the forward rate reaction equation, *D* is the diffusion constant, and is approximated by the Stokes-Einstein equation (**Equation S5**^8^), and $a_{ALK-ATP}$ is the radius of gyration of ALK-TKD (**Equation S6**^9^). In the Stokes-Einstein equation, water dynamic viscosity (η) is applied to account for diffusion of a ligand such as ATP in water, and *r* = 14 Å, which approximates ATP diameter. The ALK radius of gyration ($a_{ALK-ATP}$) is used as an approximation of ALK and ATP contact size, with N = 326 (the number of kinase domain residues). With these assumptions, *k*_on,ATP_ was estimated at 6.94 x 10^10^ M^-1^s^-1^.

$k_{on,ATP}\sim r_{f}=16 \times\pi\times D \times a_{ALK-ATP}$ **Equation S4**

$D = \frac{k_{B}T}{6\pi\eta r}$ **Equation S5**

$a_{ALK-ATP}= 3 Å \times N^{\frac{1}{3}}$ **Equation S6**

Values for the ATP dissociation rate constant (*k*_off,ATP_) for mutated and wild type ALK variants were derived from experimental values for *K*_M,ATP_ reported here (**Supplementary Table 1**), which were in turn converted to estimates of the ATP equilibrium dissociation constant (*K*_D_) using the assumptions of **Equation S7** (see **Supplementary Tables 4** and **5**).

$K_{M,ATP}=K_{D,ATP}= \frac{k_{off, ATP}}{k_{on, ATP}}$ **Equation S7**

**V. Estimation of lorlatinib association and dissociation rate constants for inhibition model**

Since we do not have experimentally-derived binding constants for lorlatinib, we used our computationally-derived docking scores to calculate dissociation rate constants, and estimated a lorlatinib association rate constant (*k*_on,lor_) for each ALK variant of 6.94 x 10^10^ M^-1^s^-1^ using the same equations employed above for estimating *k*_on,ATP_. Since the diffusion limited model in **Equation S4** only relies on size, this approximation is justified given the similar sizes for ATP and lorlatinib. Indeed, local sensitivity analysis studies showed that altering *k*_on_ by an order of magnitude does not impact the results.

Next, to calibrate our computational docking scores for lorlatinib to experimentally observed binding affinities, we compared calculated and experimental for the F1174L and R1275Q variants to derive a conversion factor (α factor) for use in comparing computational results with experimental results for the compound ALK mutations. We derived the α factor as follows:

- First, computational lorlatinib-docking scores were converted to pseudo equilibrium dissociation constants [*K*_D,lor_comp_] by applying the Arrhenius equation with ∆G representing the docking score (**Equation S8**, **Supplementary Table 4**).

$K_{D,lor\_comp}= e^{\frac{{\Delta G}^{docking score}}{KT}}= \frac{k_{offlor\_comp}}{k_{on,lor\_comp}}$ **Equation S8**

- Next, experimental equilibrium dissociation constants for lorlatinib [*K*_D,lor_exp_] were approximated by modeling ALK inhibition of F1174L and R1275Q variants using the experimental *K*_M,ATP_ values presented in **Supplementary Table 1**, and fitting the *k*_off,lor_ value that returns a lorlatinib IC_50_ value that matches the experimental result (with *k*_on,lor_ = 6.94 x 10^10^ M^-1^s^-1^). To do this, the open-gate kinetic reaction model in **Extended Data Fig. 7c** was used, with known values for *K*_M,ATP_ (experimental) and *k*_on,lor_ (6.94 x 10^10^ M^-1^s^-1^) – with ATP at 1 mM and ALK-TKD at 5 nM. The best-fit *k*_off,lor_ value was taken as *k*_off,lor_exp_ (**Supplementary Table 4**), and was used (with the estimated *k*_on,lor_) to derive a value for *K*_D,lor_exp_ using **Equation S9**.

$K_{D,lor\_exp}= \frac{k_{off,lor\_exp}}{k_{on,lor}}$ **Equation S9**

- The pseudo computational estimate of *K*_D,lor_exp_ was then related to the experimental estimate as follows. Values for *K*_D,lor_comp_ and *K*_D,lor_exp_ were related using **Equation S10** to calculated an ⍺ factor that accounts for the difference between calculation and experiment. The geometric mean of α factors for the ALK variants F1174L and R1275Q was 216.62. The α factor values were used to estimate expected *k*_off,lor_exp_ values for ALK variants based on calculated docking scores, and to estimated modeled IC_50_ values in the next Section.

$k_{off,lor\_comp}= \alpha\times\frac{k_{off,lor\_exp}}{k_{on,lor}}$ **Equation S10**

**VI. Estimation of *in silico* lorlatinib IC_50_ values for compound ALK mutations**

The mode of resistance due to the compound mutations was assessed by modeling lorlatinib IC_50_ using the kinetic models outlined in **Extended Data Fig. 7c**, with- or without gate closure. The model replicated the reaction conditions used in biochemical assays described in this paper (ATP concentration of 1 mM and ALK-TKD concentration of 5 nM.

Rates of switching between the E1 (gate-open) and E2 (gate-closed) conformations (k_1_/k_-1_) were calculated based on the percent of time ALK was seen to spend in the gate-open and gate-closed conformations (summarized in **Figure 6F**)**.** The k_1_ rate was set to 10 ns because the autocorrelation time for gate switching corresponds to the diffusion timescale of disordered loops and secondary structure elements, which occurs over timescales slower than 10 ns. The value for k_-1_ was then calculated using the ratio of residence times in the gate-open and gate-closed conformations (**Equation S11**).

$k_{-1}=k_{1}\times\frac{\% time open conformation}{\% time closed conformation}$ **Equation S11**

Reactions modeled for each complex, and the parameter values used are listed in **Supplementary Table 6**. IC_50_ values for each ALK variant were derived by varying the lorlatinib concentration with the reaction at steady state. IC_50_ was calculated and plotted by fitting the response as determined by calculated [ALK-ATP] using the Nonlinear Regression Fit [inhibitor] vs. response (three parameters) analysis tool in Graphpad Prism 9.3.1.

**Supplementary Table 1: Biochemical analysis of lorlatinib and crizotinib inhibition and ATP binding to ALK-TKD variants using purified, dephosphorylated ALK-TKD.**

*****Calculated according to Cha^2^: IC_50_ = (E_t_/2 + *K*_i_) + (*K*_i_ x [ATP])/*K*_M,ATP_ at [ATP] = 1 mM and E_t_ (total [enzyme] of 5 nM.

IC_50_ and *K*_M,ATP_ measurements are reported as mean ± SD of three independent biological experiments.

|  | **lorlatinib**  **IC_50_ (nM)** | **crizotinib**  **IC_50_ (nM)** | **lorlatinib**  ***K*_i_^*^ (nM)** | **crizotinib**  ***K*_i_^*^ (nM)** | ***K*_M,ATP_  (µM)** | ***k*_cat,app_ / *K*_M,ATP_  (min^-1^mM^-1^)** |
| --- | --- | --- | --- | --- | --- | --- |
| F1174L | 2.3 ± 1.1 | 40 ± 20 | <0.03 | 1.02 | 28 ± 4 | 1821 |
| F1174L/G1202R | 26 ± 16 | 437 ± 175 | 0.88 | 16.5 | 39 ± 21 | 744 |
| F1174L/L1196M | 12 ± 6.2 | 150 ± 51 | 0.37 | 5.81 | 41 ± 11 | 2512 |
|  |  |  |  |  |  |  |
| R1275Q | 2.9 ± 0.8 | 38 ± 24 | <0.03 | 2.57 | 78 ± 18 | 590 |
| R1275Q/G1202R | 40 ± 21 | 155 ± 89 | 1.95 | 7.95 | 55 ± 23 | 455 |
| R1275Q/L1196M | 8 ± 5.3 | 209 ± 50 | 0.45 | 17.22 | 91 ± 29 | 1220 |
|  |  |  |  |  |  |  |

**Supplementary Table 2: Distances between different carbon atoms of R1202 and L1122 at different time points in a 100 ns MD simulation of the F1174L/G1202R variant, as depicted in Extended Data Fig. 7e.**

| **Time (ns)** | **110** | **120** | **130** | **140** | **150** | **160** | **170** | **180** | **190** | **200** |
| --- | --- | --- | --- | --- | --- | --- | --- | --- | --- | --- |
| **Cα-Cα dist (Å)** | 9.75 | 10.37 | 6.58 | 7.56 | 7.51 | 7.09 | 6.69 | 7.44 | 7.86 | 9.13 |
| **Cα-Cζ dist (Å)** | 8.15 | 10.41 | 7.08 | 7.56 | 7.30 | 7.51 | 6.73 | 4.13 | 3.78 | 6.20 |
| **Cγ-Cζ dist (Å)** | 8.00 | 9.78 | 7.31 | 7.20 | 7.72 | 8.19 | 7.26 | 5.06 | 4.62 | 5.88 |
| **docking score (kcal/mol)** | -12.03 | -10.32 | -3.76 | -4.32 | -3.74 | -4.91 | -3.51 | -2.92 | -4.91 | -10.53 |

**Supplementary Table 3: Summary of structural characteristics of ALK-TKD variants during 100 ns MD simulations (n = 3), listing parameters for the lorlatinib binding site and behavior of residue 1202.**

| **ALK-TKD Variant** | **Closed Gate Presence?** | **Average Distance (Å)**  **G1202R:C⍺ to L1122:C⍺** | **Binding Pocket SASA Distribution** |
| --- | --- | --- | --- |
| **L1196M** | No | 10.8 | unimodal |
| **G1202R** | Yes | 7.5 | bimodal |
| **L1196M/G1202R** | No | 9.9 | unimodal |
| **R1275Q/G1202R** | No | 9.8 | unimodal |
| **R1275Q/L1196M** | No | 9.7 | unimodal |
| **F1174L/G1202R** | Yes | 7.6 | bimodal |
| **F1174L/L1196M** | No | 9.8 | unimodal |

**Supplementary Table 4: Kinetic constants for computational studies, derived from experimental data in Supplementary Table 1 – for calibration of computational docking scores and experimental IC_50_ measurements.**

|  | **F1174L** | **R1275Q** |
| --- | --- | --- |
| **IC_50_ lorlat (nM)** | 2.3 | 2.9 |
| ***K*_D_ (exp) ATP (mM)** | 0.028 | 0.078 |
| ***k*_off,ATP_ (s^-1^)** | 1.94 x 10^6^ | 5.41 x 10^6^ |
| ***k*_off,lor_exp_ (s^-1^)** | 0.5 | 3.0 |

**Supplementary Table 5: Calculated lorlatinib equilibrium association (*K*_A_) and dissociation (*K*_D_) constants using Equation S8.**

| **ALK variant** | ***K*_A_ (M^-1^)** | ***K*_D_ (M)** |
| --- | --- | --- |
| **wild type** | 9.99 x 10^6^ | 1.00 x 10^-7^ |
| **F1174L** | 5.24 x 10^6^ | 1.91 x 10^-7^ |
| **F1174L/G1202R** | 3.36 x 10^6^ | 2.97 x 10^-7^ |
| **F1174L/L1196M** | 7.91 x 10^5^ | 1.26 x 10^-6^ |
| **R1275Q** | 6.02 x 10^7^ | 1.66 x 10^-8^ |
| **R1275Q/G1202R** | 5.17 x 10^6^ | 1.93 x 10^-7^ |
| **R1275Q/L1196M** | 9.23 x 10^6^ | 1.08 x 10^-7^ |

**Supplementary Table 6: Calculated rates of each reaction in Extended Data Fig. 7c for each ALK-TKD variant.**

| **Reactions** | **Rates** | **F1174** | **F1174/ G1202R** | **F1174/ L1196M** | **R1275Q** | **R1275Q/ G1202R** | **R1275Q/ L1196M** |
| --- | --- | --- | --- | --- | --- | --- | --- |
| E1 + A → E1A | *k*_on,ATP_ (M/s^-1^) | 6.94 x 10^10^ | 6.94 x 10^10^ | 6.94 x 10^10^ | 6.94 x 10^10^ | 6.94 x 10^10^ | 6.94 x 10^10^ |
| E1A → E1 + A | *k*_off,ATP_ (s^-1^) | 1.94 x 10^6^ | 2.71 x 10^6^ | 2.84 x 10^6^ | 5.41 x 10^6^ | 3.81 x 10^6^ | 6.31 x 10^6^ |
| E2 + A → E2A | *k*_on,ATP_ (Ms^-1^) | 6.94 x 10^10^ | 6.94 x 10^10^ | 6.94 x 10^10^ | 6.94 x 10^10^ | 6.94 x 10^10^ | 6.94 x 10^10^ |
| E2A → E2 + A | *k*_off,ATP_ (s^-1^) | 1.94 x 10^6^ | 2.71 x 10^6^ | 2.84 x 10^6^ | 5.41 x 10^6^ | 3.81 x 10^6^ | 6.31 x 10^6^ |
| E1 + L → E1L | *k*_on,lor_ (Ms^-1^) | 6.94 x 10^10^ | 6.94 x 10^10^ | 6.94 x 10^10^ | 6.94 x 10^10^ | 6.94 x 10^10^ | 6.94 x 10^10^ |
| E1L → E1 + L | *k*_off,lor_comp_ (s^-1^) | 61.09 | 95.24 | 405.09 | 5.32 | 61.92 | 34.69 |
| E1 → E2 | k_1_ (s^-1^) | 1.00 x 10^8^ | 1.00 x 10^8^ | 1.00 x 10^8^ | 1.00 x 10^8^ | 1.00 x 10^8^ | 1.00 x 10^8^ |
| E2 → E1 | k_-1_ (s^-1^) | 5.78 x 10^9^ | 1.45 x 10^7^ | 3.35 x 10^8^ | 5.67 x 10^8^ | 3.84 x 10^8^ | 3.41 x 10^8^ |
| E1 + L → E1L | *k*_off,lor_comp_ (s^-1^)^†^ | 68.69 | 4.07 | 24.04 | 3.64 | 11.54 | 45.07 |
| E1 + L → E1L | *k*_off,lor_comp_ (s^-1^)^‡^ | 54.33 | 2227.83 | 6826.21 | 7.77 | 332.06 | 26.71 |

† Derived based on the upper SD of docking scores in Cluster 1

‡ Derived based on the lower SD of docking scores in Cluster 1.

**Supplementary References.**

1. Pantsar, T. & Poso, A. Binding affinity via docking: Fact and fiction. *Molecules* **23**, 1899 (2018).

2. Cha, S. Tight-binding inhibitors-I. Kinetic behavior. *Biochem. Pharmacol.* **24**, 2177-2185 (1975).

3. Infarinato, N.R.*, et al.* The ALK/ROS1 inhibitor PF-06463922 overcomes primary resistance to crizotinib in ALK-driven neuroblastoma. *Canc. Discov.* **6**, 96-107 (2016).

4. Newton, P., Harrison, P. & Clulow, S. A novel method for determination of the affinity of protein: protein interactions in homogeneous assays. *J. Biomol. Screen* **13**, 674-682 (2008).

5. Pronk, S.*, et al.* GROMACS 4.5: a high-throughput and highly parallel open source molecular simulation toolkit. *Bioinformatics* **29**, 845-854 (2013).

6. Johnson, T.W.*, et al.* Discovery of (10R)-7-amino-12-fluoro-2,10,16-trimethyl-15-oxo-10,15,16,17-tetrahydro-2H-8,4-(metheno)pyrazolo[4,3-h][2,5,11]-benzoxadiazacyclotetradecine-3-carbonitrile (PF-06463922), a macrocyclic inhibitor of anaplastic lymphoma kinase (ALK) and c-ros oncogene 1 (ROS1) with preclinical brain exposure and broad-spectrum potency against ALK-resistant mutations. *J. Med. Chem.* **57**, 4720-4744 (2014).

7. Hoops, S.*, et al.* COPASI--a COmplex PAthway SImulator. *Bioinformatics* **22**, 3067-3074 (2006).

8. Dill, K.A. & Bromberg, S. Molecular driving forces: Statistical thermodynamics in biology, chemistry, physics, and nanoscience. (Garland Science, London and New York, 2011).

9. Gong, H., Fleming, P.J. & Rose, G.D. Building native protein conformation from highly approximate backbone torsion angles. *Proc. Natl. Acad. Sci. U.S.A.* **102**, 16227-16232 (2005).
